# 维奈克拉联合减低剂量HAD方案诱导治疗急性髓系白血病的疗效观察

**DOI:** 10.3760/cma.j.cn121090-20231208-00298

**Published:** 2024-04

**Authors:** 嶂松 阎, 洋 李, 斌 张, 劲松 贺, 捷思 李, 述宁 魏, 齐 王, 秋玲 李, 凯奇 刘, 营昌 秘

**Affiliations:** 1 中国医学科学院血液病医院(中国医学科学院血液学研究所)，血液与健康国家重点实验室，国家血液系统疾病临床医学研究中心，细胞生态海河实验室，天津 300020 State Key Laboratory of Hematology and Health, National Clinical Research Center for Blood Diseases, Haihe Laboratory of Cell Ecosystem. Institute of Hematology & Blood Diseases Hospital, Chinese Academy of Medical Sciences & Peking Union Medical College, Tianjin 300020, China; 2 天津医学健康研究院，天津 301600 Tianjin Medical Health Research Institute, Tianjin 301600, China; 3 天津市中西医结合医院，天津市南开医院，天津 300102 Tianjin Hospital of ITCWM, Nankai Hospital, Tianjin 300102, China

## Abstract

通过回顾性分析自2022年5月至2023年1月收治的25例初诊急性髓系白血病（AML）患者，探索应用维奈克拉联合减低剂量HAD（高三尖杉酯碱+柔红霉素+阿糖胞苷）方案诱导治疗初诊AML的疗效及安全性。按照ELN 2017预后分层标准，低危组13例（52.0％），中危组3例（12.0％），高危组9例（36.0％）。1个疗程诱导治疗总体有效率（完全缓解率+部分缓解率）88.0％，1个疗程完全缓解率84.0％。截至2023年5月30日，中位随访9个月，全部患者诱导治疗后给予巩固治疗2（1～5）个周期。1年总生存率、无事件生存率、无复发生存率分别为100％、94.7％、94.7％。维奈克拉联合减低剂量HAD诱导方案，1个疗程完全缓解率高，安全性良好。

## Abstract (en)

The efficacy and safety of venetoclax combined with reduced dose HAD regimen in the treatment of newly diagnosed acute myeloid leukemia（AML）was investigated. From May 2022 to January 2023, a total of 25 patients with newly diagnosed AML were treated with venetoclax combined with reduced-dose HAD regimen as induction therapy. Accoding to the 2017 ELN recommendations, 13（52.0％）in favoable, 3（12.0％）in intemediate, and 9（36.0％）in adverse. The ORR（CR rate+PR rate）was 88.0％, and the CR rate was 84.0％. By May 30, 2023, with a median follow-up of 9 months, 1 year overall survival, event-free survival, and relapse-free survival were 100％, 94.7％, and 94.7％, respectively. All patients received 1–5 cycles of consolidation therapy and two median cycles. Treatment with venetoclax and reduced dose of HAD regimen in the treatment of patients with newly diagnosed AML was high effective and safe.

BCL-2抑制剂维奈克拉（VEN）的应用显著改善了急性髓系白血病（AML）患者的疗效[1]。VEN联合阿扎胞苷（AZA）、地西他滨和小剂量阿糖胞苷（Ara-C）等显著提高了不适合强化疗的初治AML患者的疗效和预后[2]–[4]；而VEN和传统化疗方案（IA、CLIA、FLAG-IDA、DA）联合也显著提高了适合强化疗的初诊AML患者的完全缓解（CR）率和微小残留病（MRD）转阴率，改善了患者的生存[5]–[9]。以高三尖杉酯碱（HHT）联合Ara-C为基础的方案是我国成人AML（非急性早幼粒细胞白血病）诊断和治疗指南推荐的诱导治疗方案之一[10]，HHT联合柔红霉素（DNR）及Ara-C（HAD）诱导治疗初治AML患者1个疗程CR率为60％～70％[11]–[13]。为探索适合强化疗的AML患者的缓解率高、不良反应小的治疗方案，我们采用VEN联合减低剂量的HAD方案进行诱导治疗，结果报道如下。

## 病例与方法

一、病例

回顾性分析2022年5月至2023年1月我中心收治的25例新诊断的成人AML患者（16～60岁），美国东部肿瘤协作组（ECOG）评分<2分。诊断分型采用2016年WHO AML诊断标准。

二、治疗方案

1. 诱导治疗：VEN 100 mg第−1天，200 mg第0天，400 mg，第1～7天；HHT 2 mg·m^−2^·d^−1^，第2～6天；DNR 45 mg·m^−2^·d^−1^，第4～5天；Ara-C 100 mg·m^−2^·d^−1^，第2～6天。

2. 缓解后治疗：达到CR的患者根据欧洲白血病网络（ELN）指南（2017年版）和中国成人AML诊治指南（2021年版）的推荐进行巩固治疗4～5个疗程，巩固治疗包括2个周期VEN联合中剂量Ara-C（VEN 400 mg/d第1～7天；Ara-C 1 g·m^−2^·12 h^−1^，第2～4天），2个周期DA方案（DNR 45 mg·m^−2^·d^−1^，第1～2天；Ara-C 100 mg·m^−2^·d^−1^，第1～5天），1个周期HA方案（HHT 2 mg/m^2^，第1～5天；Ara-C 100 mg·m^−2^·d^−1^，第1～5天）。适合行异基因造血干细胞移植（allo-HSCT）的患者巩固治疗3～4个疗程后进入移植流程。不需要或者不能进行allo-HSCT的患者继续接受维持治疗。维持治疗方案为AZA 100 mg/d，第1～5天，每月1个周期，共4～6个周期。

三、疗效评价标准

主要研究目的是评价总体有效率、总生存（OS）时间、无病生存（DFS）时间及无事件生存（EFS）时间，参考ELN 2017疗效标准[14]进行。

四、随访

随访截至2023年5月20日或失访日期（失访者），中位随访时间9（3～12）个月。EFS时间指自诊断日到第1次事件（包括未缓解、分子生物学和临床复发、CR期间的死亡）发生或末次随访日期。DFS时间指CR至白血病复发或在CR期间死亡或末次随访日期。OS时间指自诊断日至死亡或末次随访日期。

五、统计学处理

统计学分析采用 SPSS v.25.0软件。采用精确二项法计算有效率及其95％可信区间。应用Kaplan-Meier生存曲线和Log-Rank检验计算OS、EFS和RFS率及其95％ *CI*。

## 结果

1. 患者临床特征：共纳入25例初诊AML患者，临床特点见表1。其中男16例，女9例；中位年龄34（17～58）岁。患者初诊时中位WBC 14.09（1.85～114.55）×10^9^/L。按照ELN2022预后分层，其中预后良好组13例（52.0％）、中等组3例（12.0％）和不良组9例（36.0％）。

**表1:** 25例初诊急性髓系白血病患者临床资料

项目	数值
年龄[岁，*M* (范围)]	34 (17~58)
性别[例（%）]	
男	16 (64.0)
女	9 (36.0)
ECOG评分[分，*M* (范围)]	1 (0~2)
WBC [×10^9^/L，*M* (IQR)]	14.09 (1.85~114.55)
PLT[×10^9^/L，*M* (IQR)]	54 (9~211)
ELN 2022风险分组 [例（%）]	
良好	13 (52.0)
中等	3 (12.0)
不良	9 (36.0)
细胞遗传学风险分组[例（%）]	
良好	3 (12.0)
中等	18 (72.0)
不良	4 (16.0)
分子学突变 [例（%）]	
NPM1	11(44.0)
FLT3-ITD	8 (32.0)
DNMT3A	6 (24.0)
CEBPA (B-zip)	5 (20.0)
RAS	4 (16.0)
IDH2	3 (12.0)
KIT	2 (8.0)
ASXL1	2 (8.0)
IDH1	2 (8.0)
PTPN11	2 (8.0)
U2AF1	2 (8.0)
TP53	1 (4.0)
TET2	1 (4.0)

**注** ECOG：美国东部肿瘤协作组；ELN：欧洲白血病网络

2. 疗效分析：所有患者1个疗程诱导CR率84.0％（21/25例），部分缓解（PR）率4.0％（1/25例），未缓解3例，总体有效率（CR率+PR率）为88.0％（22/25例）。按照ELN2017预后标准，低危组患者CR率为92.3％（12/13），中高危组患者CR率为75.0％（9/12）。诱导治疗1个疗程达CR的患者，流式细胞术检测MRD转阴17例（81.0％）。

1个疗程诱导治疗后3例未缓解患者：1例伴有FLT3-ITD（AR：1.55）、NPM1、U2AF1和RAD1突变，经索拉非尼联合MA方案（米托蒽醌和Ara-C）挽救治疗后达CR。1例初诊时染色体核型为42-45,XY，−2，add（3），−5，del（6），add（11），−17，+1-2 mar，伴TP53突变，给予地西他滨+阿柔比星+Ara-C挽救治疗达CR。1例伴FLT3-ITD（AR:14.75）、NPM1、DNMT3A突变，予以VEN联合索拉非尼和AZA再诱导获得CR。PR患者经MAC（米托蒽醌+Ara-C+环磷酰胺）再诱导后获得CR。

3. 治疗相关不良反应：诱导治疗期间最常见的血液学不良事件为3～4级的贫血、血小板减少和中性粒细胞减少，发生于所有患者。最常见的3～4级非血液学不良事件为粒细胞缺乏期感染（100％），其次分别为肺炎（52.0％,13/25）、上呼吸道感染（28.0％,7/25）、皮肤软组织感染（20.0％,5/25）、菌血症（8.0％,2/25）、肠道感染（12.0％,3/25）、口腔感染（20.0％,5/25）。1例患者化疗第3天发生肿瘤溶解综合征，停止治疗，碱化利尿等对症支持治疗后好转。血象恢复后复查骨髓达CR。无患者在诱导治疗期间死亡。达CR患者诱导治疗后中性粒细胞计数恢复至≥0.5×10^9^/L的中位时间为13（10～18）d，PLT恢复至≥30×10^9^/L的中位时间为12（8～19）d。

4. 患者生存情况：截至2023年5月20日，中位随访9（3～12）个月，25例患者中9例（36.0％）患者行allo-HSCT（1例移植后复发），无患者死亡。预计1年OS、EFS和DFS率分别为100％、94.7％（95％ *CI* 89.6％～99.8％）和94.7％（95％ *CI* 89.6％～99.8％）。

## 讨论

近年来，随着索拉非尼、米哚妥林、吉瑞替尼、VEN等一些分子靶向药物特别是VEN在临床中的应用，明显提高了初诊AML患者的疗效[1],[15]–[17]。VEN联合阿扎胞苷或地西他滨治疗成为老年或不耐受强化疗初诊AML患者的标准诱导方案。对于适合化疗的患者，多项研究显示：VEN联合化疗明显提高了患者的诱导缓解率和缓解后MRD转阴率[5]–[9]。HAD作为我国初诊AML患者的传统诱导治疗方案，已被纳入中国成人AML治疗指南。如何把我国传统诱导方案HAD与VEN相结合用于初诊AML患者，进一步提高其疗效及缓解深度，目前尚无报道。

本研究中，我们首次采用VEN联合减低剂量HAD方案诱导治疗初诊的AML患者。研究证实，为减少VEN联合化疗对骨髓抑制的重叠作用，联合减低剂量的化疗方案也能获得良好的有效性与安全性[5]，因此本研究方案与常规HAD方案比较，HHT和Ara-C的剂量由原来的7 d减少到5 d，DNR的剂量减少了1 d。我们既往常规HAD方案诱导治疗初诊AML患者1个疗程CR率70％[11]，本研究方案诱导治疗初诊AML患者1个疗程CR率84.0％，1个疗程总体有效率为88.0％。MRD转阴率也提高至79.2％，与前期报道的VEN联合化疗疗效类似[5]–[9]。

在巩固治疗中，既往研究结果显示，含中剂量Ara-C联合方案与大剂量Ara-C方案的DFS率与OS率差异无统计学意义[11]，因此本研究中全部患者采用统一巩固方案。方案中FLT3-ITD突变患者诱导治疗未加用FLT3抑制剂。截至2023年5月30日，全部患者诱导治疗后中位给予巩固治疗2（1～5）个周期。

本组研究中，常见的血液学不良事件为4级的中性粒细胞减少（100％）和血小板减少（100％）；最常见的3～4级非血液学不良事件为感染，包括粒细胞缺乏期感染、肺炎、上呼吸道感染、菌血症、肠道感染等。1例患者出现肿瘤溶解综合征，经碱化利尿等治疗后好转。无患者诱导期死亡，显示出该联合方案良好的耐受性。值得注意的是，该诱导治疗方案骨髓抑制期较短，从停止治疗开始，中性粒细胞和血小板恢复的中位时间分别为13 d和12 d。与已报道的VEN联合化疗方案比较[5]–[7]，抑制期明显缩短，有利于控制感染。

截至2023年5月30日，中位随访9个月，25例患者1年OS、EFS和DFS率分别为100％、94.7％（95％ *CI* 89.6％～99.8％）和94.7％（95％ *CI* 89.6％～99.8％）。初步结果显示，该方案对初治AML患者的OS和EFS均有改善。

本研究结果显示VEN联合HAD方案对于初治AML患者有良好的疗效及耐受性。因患者例数较少，确切的结论仍需要进一步扩大患者数量及延长随访时间进一步验证。
